# Supplementary figures and images for: EsoDetect: computational validation and algorithm development of a novel diagnostic and prognostic tool for dysplasia in Barrett’s esophagus
Source: PeerJ. 2025 Jul 3;13:e19613. doi: 10.7717/peerj.19613 (PMC12229151; doi:10.7717/peerj.19613)

# LGDBE vs NDBE (all datasets)

**A**

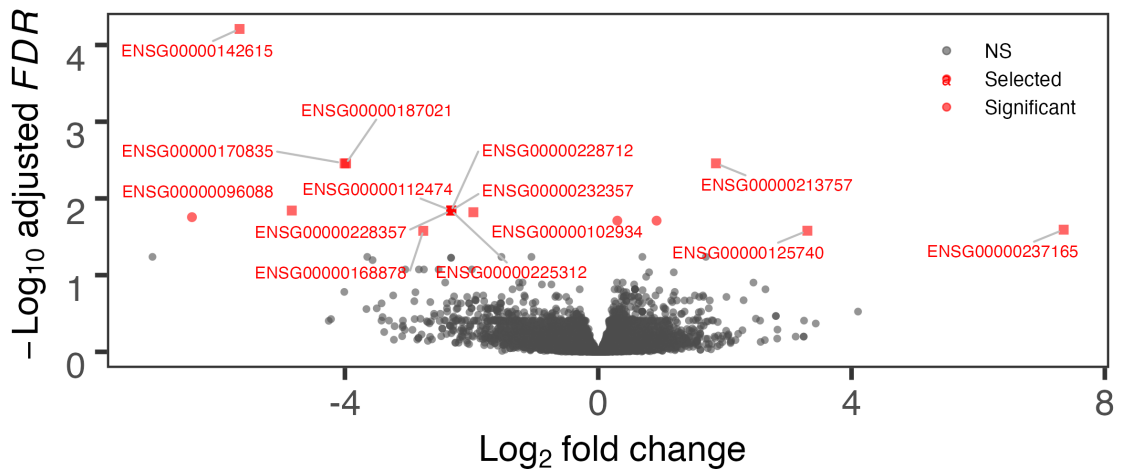

**B**

# HGDBE vs NDBE (all datasets)

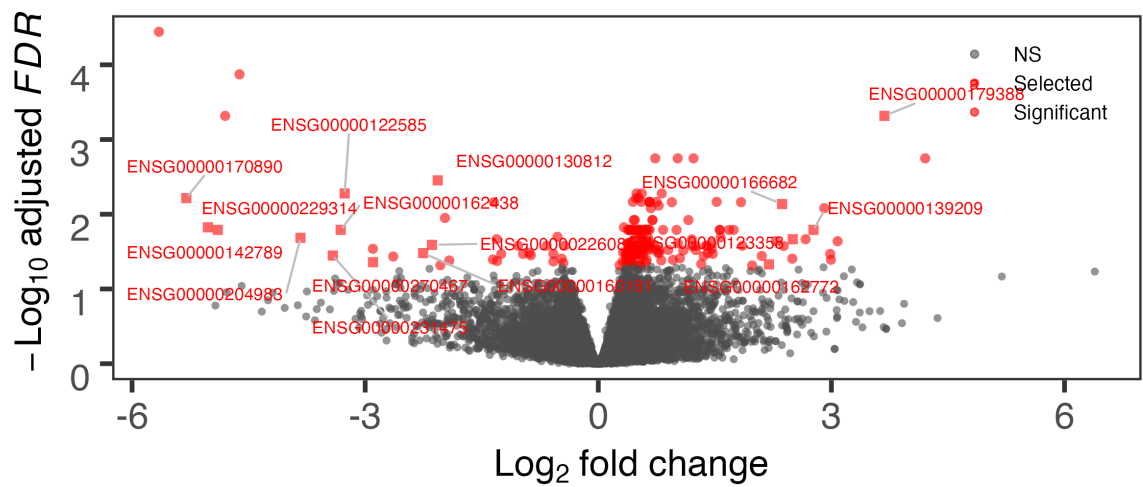

**C**

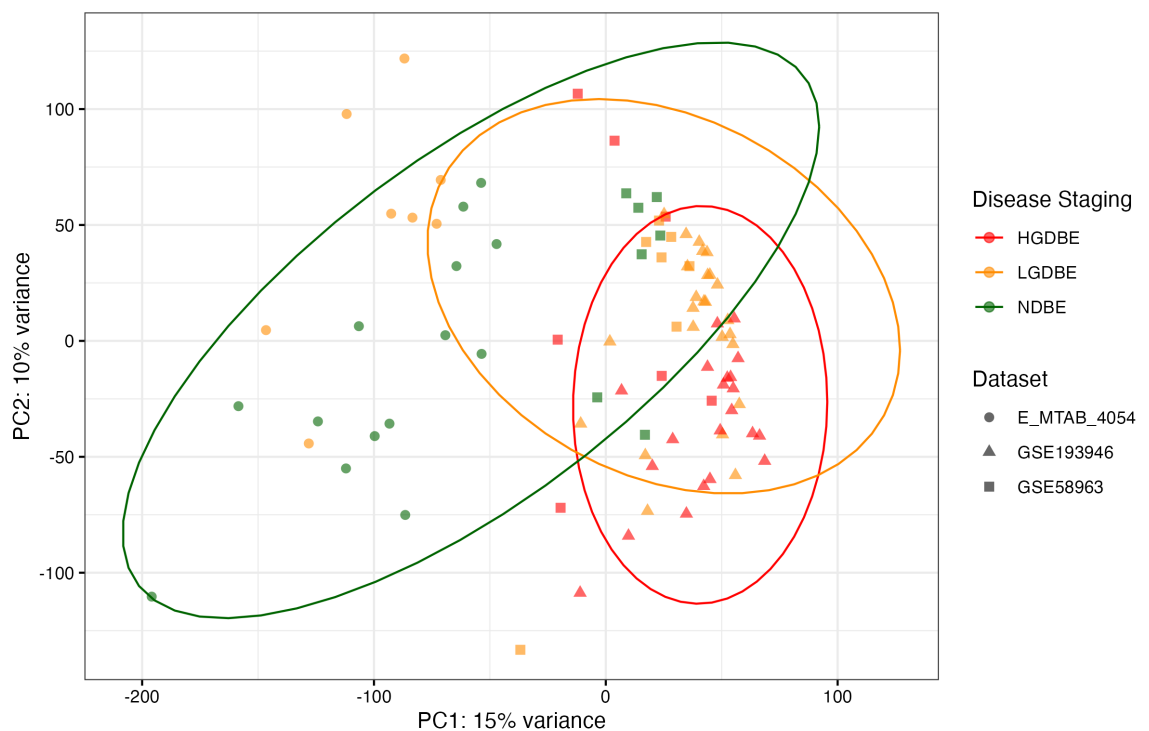

Supplement: Supplemental Information 1 [file peerj-13-19613-s001.pdf]

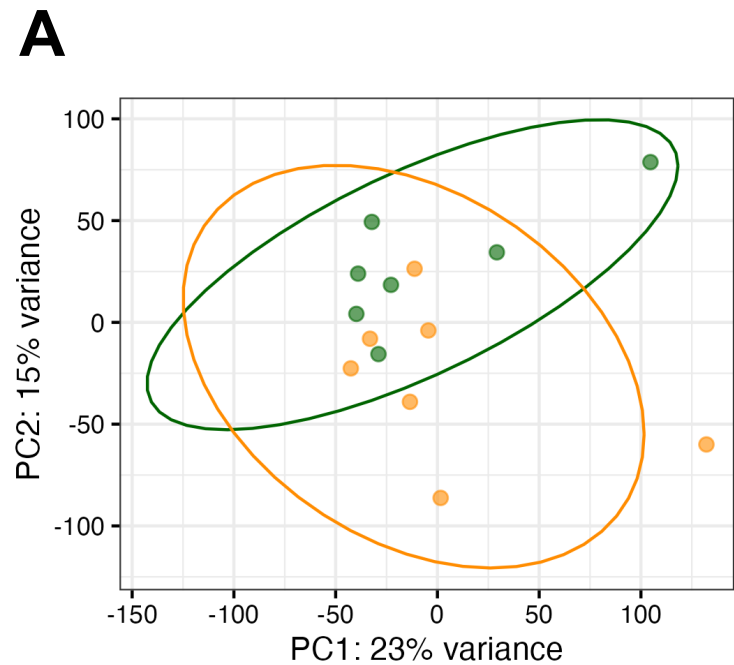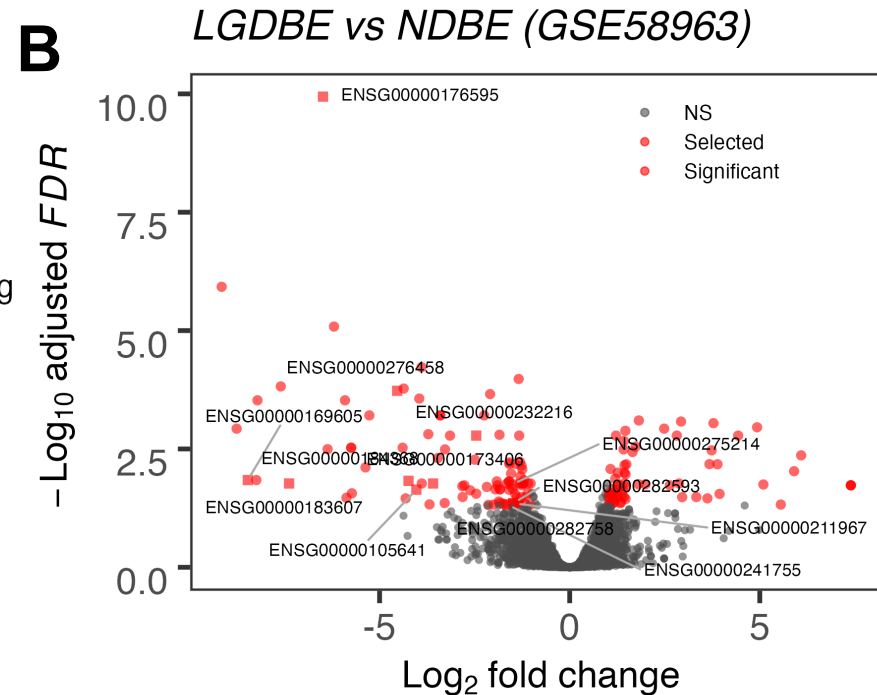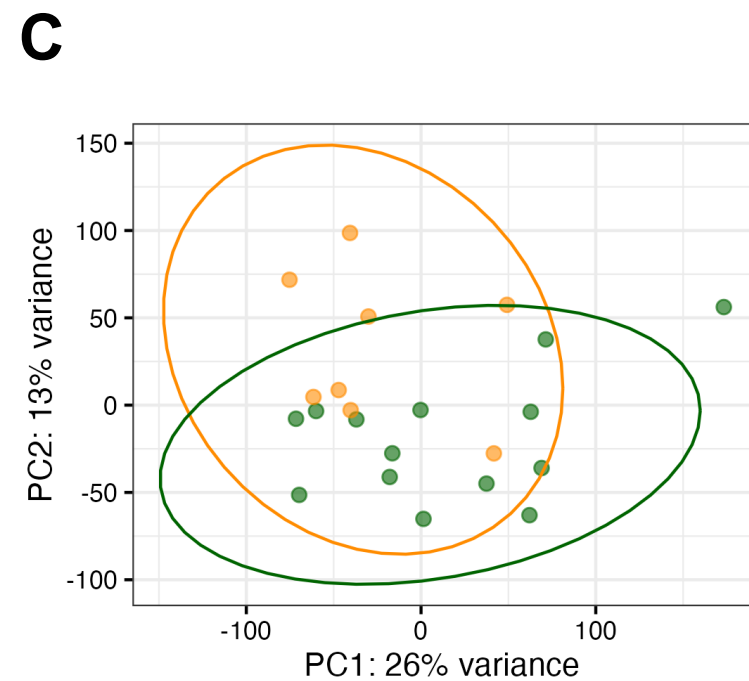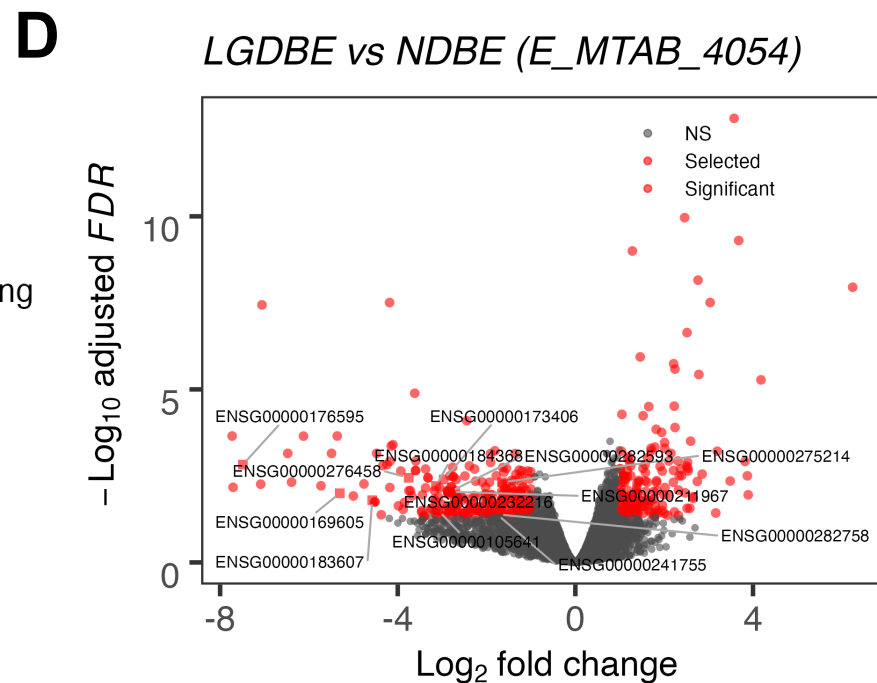

Supplement: Supplemental Information 2 [file peerj-13-19613-s002.pdf]

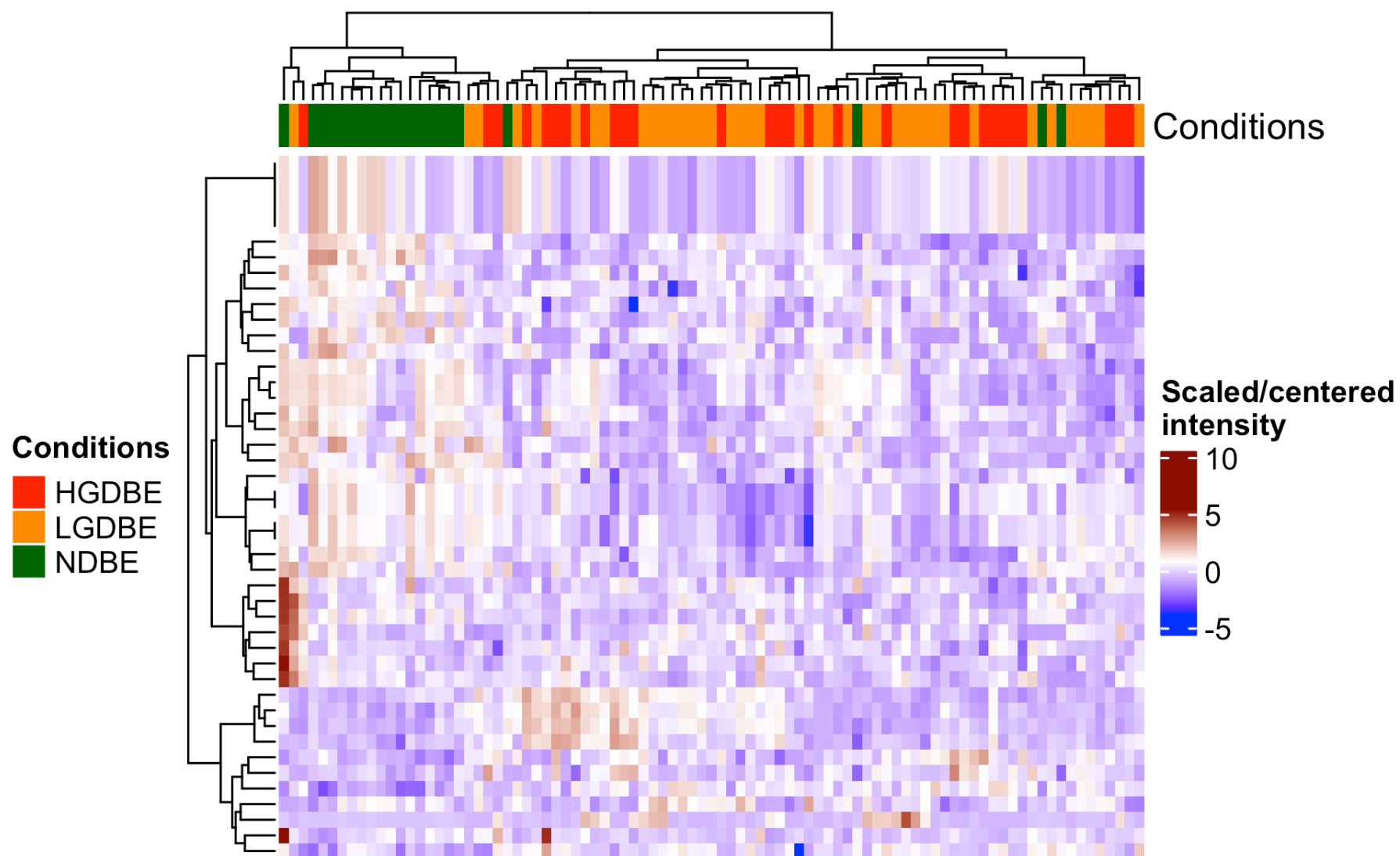

Supplement: Supplemental Information 3 [file peerj-13-19613-s003.pdf]

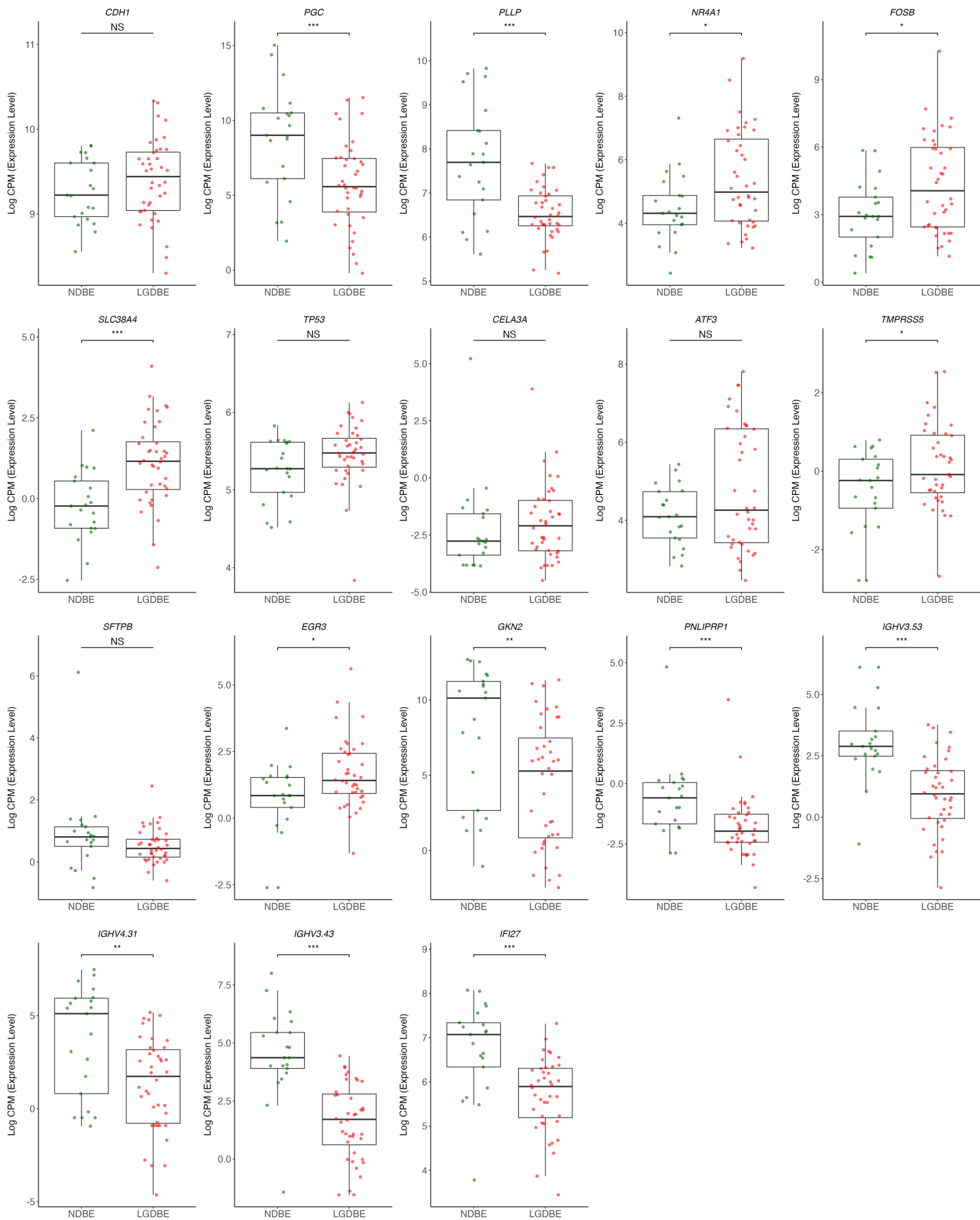

Supplement: Supplemental Information 4 — Comparison of each gene expression in NDBE and LGDBE samples obtained from a total of 3 datasets. ***adj.p < 0.001; **adj.p < 0.01; *adj.p < 0.05; NS adj.p < 0.05. [file peerj-13-19613-s004.pdf]

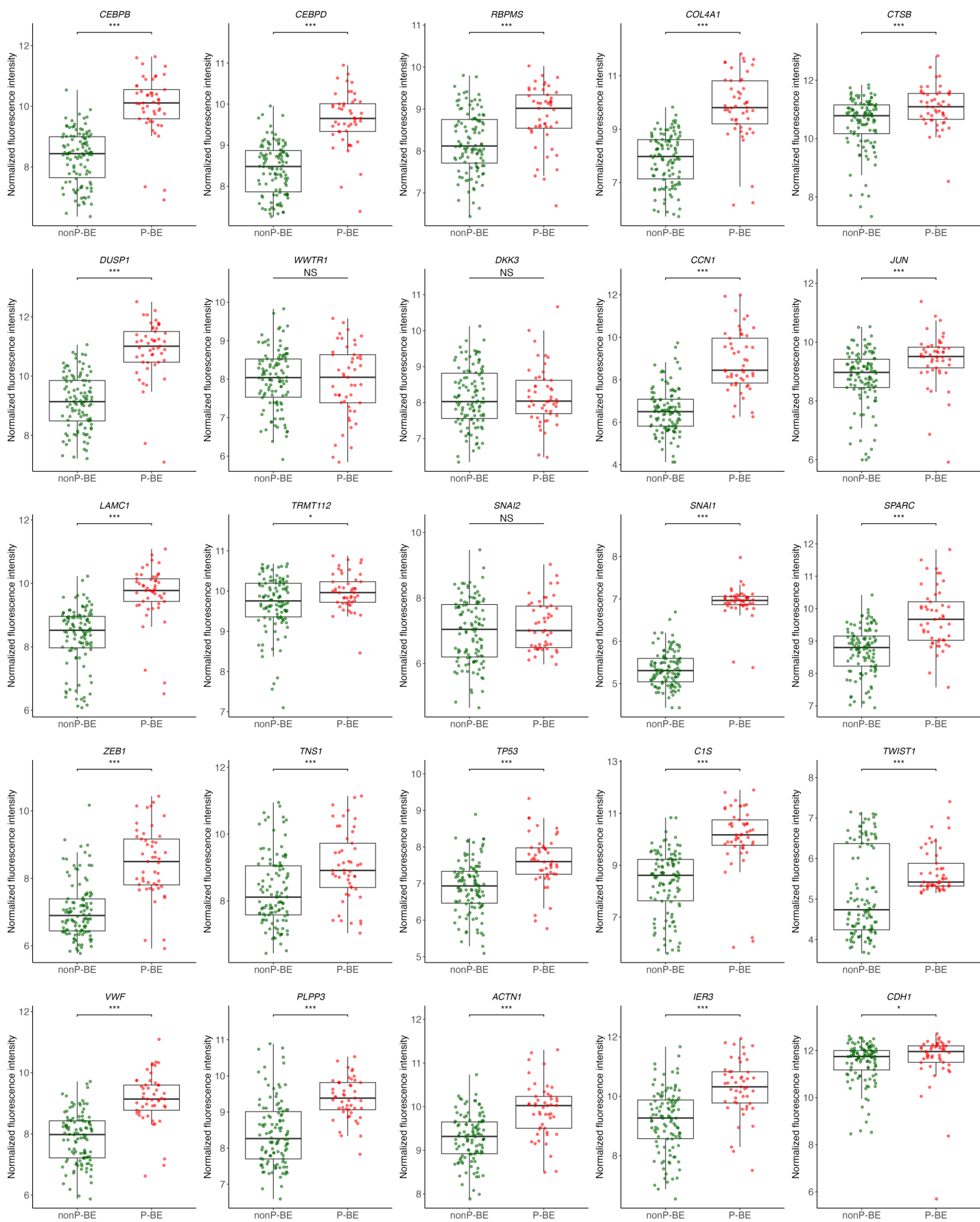

Supplement: Supplemental Information 5 — Comparison of each gene expression in nonP-BE and P-BE samples obtained from a total of 9 datasets. ***adj.p < 0.001; **adj.p < 0.01; *adj.p < 0.05; NS adj.p < 0.05. [file peerj-13-19613-s005.pdf]
