# Supplementary material for: EsoDetect: computational validation and algorithm development of a novel diagnostic and prognostic tool for dysplasia in Barrett’s esophagus
Source: PeerJ. 2025 Jul 3;13:e19613. doi: 10.7717/peerj.19613 (PMC12229151; doi:10.7717/peerj.19613)
Supplement: Supplemental Information 6 [file peerj-13-19613-s006.docx]

| **Study** | **Objective** | **Methods** | **Key Findings** | **Limitations** |
| --- | --- | --- | --- | --- |
| Spechler & Souza (2014) | Provide a review of the pathophysiology and management of BE and its progression to EAC | Analyze and integrate data from various sources to present current understanding and recommendations concerning BE | Chronic reflux is a major driver of BE development, increasing EAC risk | Does not explore molecular or genetic markers for risk stratification and to develop targeted therapies |
| Sung et al. (2021) | Provide an update on the global cancer burden estimation | Analyze the Worldwide Cancer statistics | EAC represents 2/3 of esophageal cancer in high-income countries | Projected estimates may vary depending on the region |
| Morgan et al. (2022) | Provide a global overview on the burden and prediction of esophageal cancer | Analyze the Worldwide Cancer statistics | 65% increase in EAC cases projected in 20 years | The projections are dependent on the modeling |
| Li et al. (2023) | Report the GERD prevalence Worldwide | Analyzes of the Worldwide Cancer statistics | ~783 million GERD cases globally in 2019 | There is a potential underreporting in low-resource areas |
| Lee et al. (2018) | Analyze the benefits of Seattle biopsy protocol in BE diagnosis | Integration of the Seattle protocol in existing protocol for BE diagnosis | The use of Seattle protocol improved the diagnosis of BE | Sampling errors and detection variability between professionals |
| Shaheen et al. (2022) | Revise the guidelines associated with BE and EAC diagnosis and management | Implement the GRADE methodology to propose recommendations | The use of biomarkers would improve diagnostic accuracy in the context of BE | Recommendations regarding the use of Biomarkers could not be made due to insufficient data |
| Honing & Fitzgerald (2023) | Summarize the most relevant risk factors for BE progression and  highlight the most promising novel tools for risk stratification | Review of currently available risk stratification methods | The use of p53 staining, tools like TissueCypher and molecular biomarkers combined with standard methods can improve risk stratification of BE | There are still knowledge gaps and the need of further validation for the emerging tools |
| Ross-Innes et al. (2017) | Evaluate the use of Cytosponge coupled with clinical and molecular biomarkers as a tool for BE detection | Multicentric study using Cytosponge, clinical and demographic data and a panel of biomarkers | Cytosponge combined with molecular markers can be used to determine low risk BE progression rates | Less effective for high-risk BE progression assessment |
