# Supplementary material for: EsoDetect: computational validation and algorithm development of a novel diagnostic and prognostic tool for dysplasia in Barrett’s esophagus
Source: PeerJ. 2025 Jul 3;13:e19613. doi: 10.7717/peerj.19613 (PMC12229151; doi:10.7717/peerj.19613)
Supplement: Supplemental Information 7 [file peerj-13-19613-s007.docx]

| **Target** | **Sequence** | **Expected product size (bp)** |
| --- | --- | --- |
| **Diagnostic** | | |
| **SLC38A4** | F- GTGGAGAAAGTGCTCCAGATAG  R- GGAATTTCTGACTTTCAGTGTCTTC  Probe - 56-FAM/AG CAA ATT G/ZEN/A CTG CTC ATT GCT GCC /3IABkFQ | 102 |
| **TMPRSS5** | F- CCAATCACCCAGGTGTCTAC  R- CTCAGAGGAGGGAGTCCTG  Probe - 56-FAM/AG TCC AGA A/ZEN/A CTC AGC TAC CTT GGC /3IABkFQ | 81 |
| **EGR3** | F- CGGTGGGAGAGAGAATGTAATG  R- CAAGTAGGTCACGGTCTTGTT  Probe - 56-FAM/AC TCT CTT A/ZEN/C TCC GGC TCC TTC CA/3IABkFQ | 115 |
| **TP53** | F- TCACTCCAGCCACCTGA  R- GGCCCTTCTGTCTTGAACAT  Probe - 56-FAM/TT ATG GCG G/ZEN/G AGG TAG ACT GAC CC/3IABkFQ | 78 |
| **FOSB** | F- TCCAGGCGGAGACAGAT  R- CACAAACTCCAGACGTTCCT  Probe - 56-FAM/AA AGC AGA G/ZEN/C TGG AGT CGG AGA TC/3IABkFQ | 95 |
| **NR4A1** | F- CAGGTGCAAGCCACATT  R- TACAGGGCATCTATCTAGCC  Probe - 56-FAM/AA GAC CTG C/ZEN/C TGA AGC CGG ATT /3IABkFQ | 130 |
| **IFI27** | F- CACATCCAAGCTTAAGACGGT  R- CTGTAGCAATCCTGGCCAAA  Probe - 56-FAM/AG AGT TCC T/ZEN/G AGA ATG TGA AGC TGA CC/3IABkFQ | 75 |
| **PLLP** | F- CCTGGCCACTGGTGTTAAT  R- CTCAGGGATGTCAGGTCAAC  Probe - 56-FAM/TT AAC ATC A/ZEN/G CGC CAC CGT TCT CT/3IABkFQ | 100 |
| **CELA3A** | F- TGCATCTCGAGGGATCTG  R- AGCTCCTCAGAGTTGATGG  Probe - 56-FAM/AG GTT GTA C/ZEN/T CAC CCA ACA CCA CC/3IABkFQ | 101 |
| **ATF3** | F- CTCTGCGCTGGAATCAG  R- TTTCCTTTCATCTTCTTCAGG  Probe - 56-FAM/TC ACT GTC A/ZEN/G CGA CAG ACC C/3IABkFQ | 91 |
| **CDH1** | F- GTGCTCTTCCAGGAACC  R-GAGGATGGTGTAAGCGATG  Probe - 56-FAM/AC CAA AGT C/ZEN/A CGC TGA ATA CAG TGG G/3IABkFQ | 95 |
| **GKN2** | F- GAAACAGGCTCTGGACAAC  R- GGAACCAATCCACGTCTTT  Probe - 56-FAM/CT CCA GCA A/ZEN/A TAC ACC TGG GTC AAG T/3IABkFQ | 94 |
| **IGHV3-43** | F- AAGGTGTCCAGTGTGAAGTG  R- GGACCCAGTGCATGGTATAAT  Probe - 56-FAM/TG CAG CCT C/ZEN/T GGA TTC ACC TTT GA/3IABkFQ | 126 |
| **IGHV4-31** | F- TCCCAGATGGGTCCTGTC  R- AGGGACAGGGTCTGTGAA  Probe - 56-FAM/TC CGG CTC A/ZEN/G GAC TGG TGA A/3IABkFQ | 78 |
| **IGHV3-53** | F- TCCGAGGTGTTTCCATTCG  R- CCTCACACTGGACACCTTT  Probe - 56-FAM/TG GTG AGT C/ZEN/C TCT GTG TTC AGT GC/3IABkFQ | 110 |
| **PGC** | F- GCAGCAGTGGTCAAAGTG  R- GGTACTTCCAAGCAGGATCATA  Probe - 56-FAM/AG CAA GCC C/ZEN/T TCT CCT TCA TGG TC/3IABkFQ | 118 |
| **Prognostic** | | |
| **COL4A1** | F- AGTGCCAGGCCCTCAAG  R- TTGAGCCGCAAGTCGAAATAAA  Probe - 56-FAM/TG GCC TAT C/ZEN/A GCC CTG GTG TAC /3IABkFQ | 90 |
| **LAMC1** | F- CTGGGGGCAACGTGGCCTTTT  R- CCATTCCTGCAGCACAGGGCTATT  Probe - 56-FAM/AA GGC CCA G/ZEN/C GCC TAT AAC TTT GA/3IABkFQ | 83 |
| **CEBPB** | F- GGGACCCAGCATGTCTC  R- CCGCCTCGTAGTAGAAGTT  Probe - 56-FAM/CG CCG CCT G/ZEN/C CTT TAA ATC CAT G/3IABkFQ | 81 |
| **CCN1** | F- GAAGCGGCTCCCTGTTT  R- CTGGGACCATGAAGTTGTTTG  Probe - 56-FAM/AG CCT CGC A/ZEN/T CCT ATA CAA CCC TT/3IABkFQ | 94 |
| **SNAI1** | F- CTATGCCGCGCTCTTTC  R- CTGGAAGGTAAACTCTGGATTAG  Probe - 56-FAM/AA TCG GAA G/ZEN/C CTA ACT ACA GCG AGC /3IABkFQ | 98 |
| **C1S** | F- AGAGTTCCAAGTCCCATACA  R- TTTATGTCTGTGGCAACATAGT  Probe - 56-FAM/AC AAA CTC C/ZEN/A GGT GAT CTT TAA GTC AGA CT/3IABkFQ | 108 |
| **ZEB1** | F- CCTGTGCAGTTACACCTTT  R- CGTCACATGTCTTTGATCTCT  Probe - 56-FAM/AC AGA ACC C/ZEN/A ACT TGA ACG TCA CAT GA/3IABkFQ | 88 |
| **CEBPD** | F- GAGAGCGCCATCGACTT  R- GTGATTGCTGTTGAAGAGGTC  Probe - 56-FAM/AG CGC CTA C/ZEN/A TCG ACT CCA TGG /3IABkFQ | 105 |
| **DUSP1** | F- CTCAAAGGAGGATACGAAGC  R- CTGTCAGGGACGCTAGTA  Probe - 56-FAM/TC GAC TGT T/ZEN/T GCT GCA CAG CTC C/3IABkFQ | 107 |
| **VWF** | F- TTTGCCAGGGACCCTTTG  R- CCCATCAAAGGTGTTGACGA  Probe - 56-FAM/AA GGA ACT C/ZEN/G CGG CAG GTC AT/3IABkFQ | 97 |
| **PLPP3** | F- AACAACAACCCGAGGAGGAG  R- GAGGCCCGCCATGAAGA  Probe - 56-FAM/TG CTC ATC T/ZEN/G CCT CGA CCT CTT CT/3IABkFQ | 81 |
| **ACTN1** | F- TCAACGCCCGTTGCCAAAAGA  R- GCAGTTTCTCGGTCCGCTCC  Probe - 56-FAM/TT CCC TTC G/ZEN/C TTC TGA GTT AGG GC/3IABkFQ | 96 |
