# Supplementary material for: EsoDetect: computational validation and algorithm development of a novel diagnostic and prognostic tool for dysplasia in Barrett’s esophagus
Source: PeerJ. 2025 Jul 3;13:e19613. doi: 10.7717/peerj.19613 (PMC12229151; doi:10.7717/peerj.19613)
Supplement: Supplemental Information 12 [file peerj-13-19613-s012.docx]

| N | **Gene** | **Threshold** | **F1-score** | **Recall** | **Precision** | **Specificity** | **NPV** | **Accuracy** | **FPR** | **TP** | **FP** | **TN** | **FN** |
| --- | --- | --- | --- | --- | --- | --- | --- | --- | --- | --- | --- | --- | --- |
| 1 | *COL4A1* | 9.34 | 0.84 | 0.75 | 0.94 | 0.98 | 0.89 | 0.91 | 0.02 | 49 | 3 | 132 | 16 |
| 2 | *LAMC1* | 9.19 | 0.84 | 0.88 | 0.81 | 0.90 | 0.94 | 0.90 | 0.10 | 57 | 13 | 122 | 8 |
| 3 | *CEBPB* | 9.75 | 0.84 | 0.80 | 0.88 | 0.95 | 0.91 | 0.90 | 0.05 | 52 | 7 | 128 | 3 |
| 4 | *CCN1* | 7.68 | 0.84 | 0.88 | 0.80 | 0.90 | 0.94 | 0.89 | 0.10 | 57 | 14 | 121 | 8 |
| 5 | *SNAI1* | 6.61 | 0.82 | 0.97 | 0.71 | 0.81 | 0.98 | 0.86 | 0.19 | 63 | 26 | 109 | 2 |
| 6 | *C1S* | 9.65 | 0.83 | 0.83 | 0.83 | 0.92 | 0.92 | 0.89 | 0.08 | 54 | 11 | 124 | 11 |
| 7 | *ZEB1* | 7.66 | 0.80 | 0.91 | 0.72 | 0.83 | 0.95 | 0.86 | 0.17 | 59 | 23 | 112 | 6 |
| 8 | *CEBPD* | 9.32 | 0.81 | 0.83 | 0.79 | 0.90 | 0.92 | 0.88 | 0.10 | 54 | 14 | 121 | 11 |
| 9 | *DUSP1* | 10.46 | 0.81 | 0.82 | 0.80 | 0.90 | 0.91 | 0.88 | 0.10 | 53 | 13 | 122 | 12 |
| 10 | *VWF* | 8.88 | 0.77 | 0.80 | 0.74 | 0.87 | 0.90 | 0.85 | 0.13 | 52 | 18 | 117 | 13 |
| 11 | *TWIST* | 5.15 | 0.67 | 1.00 | 0.51 | 0.53 | 1.00 | 0.69 | 0.47 | 65 | 63 | 72 | 0 |
| 12 | *PLPP3* | 8.68 | 0.68 | 0.94 | 0.53 | 0.60 | 0.95 | 0.71 | 0.40 | 61 | 54 | 81 | 4 |
| 13 | *ACTN1* | 9.82 | 0.73 | 0.74 | 0.72 | 0.86 | 0.87 | 0.82 | 0.14 | 48 | 19 | 116 | 17 |
| 14 | *CDH1* | 5.70 | 0.50 | 1.00 | 0.33 |  |  | 0.33 | 1.00 | 65 | 135 | 0 | 0 |
| 15 | *TP53* | 7.12 | 0.62 | 0.89 | 0.48 | 0.53 | 0.91 | 0.65 | 0.47 | 58 | 64 | 71 | 7 |
