# Supplementary material for: EsoDetect: computational validation and algorithm development of a novel diagnostic and prognostic tool for dysplasia in Barrett’s esophagus
Source: PeerJ. 2025 Jul 3;13:e19613. doi: 10.7717/peerj.19613 (PMC12229151; doi:10.7717/peerj.19613)
Supplement: Supplemental Information 13 [file peerj-13-19613-s013.docx]

| N | **Gene** | **Threshold** | **F1-score** | **Recall** | **Precision** | **Specificity** | **NPV** | **Accuracy** | **FPR** | **TP** | **FP** | **TN** | **FN** |
| --- | --- | --- | --- | --- | --- | --- | --- | --- | --- | --- | --- | --- | --- |
| 1 | *SLC38A4* | -0.21 | 0.84 | 0.90 | 0.78 | 0.52 | 0.73 | 0.77 | 0.48 | 36 | 10 | 11 | 4 |
| 2 | *TMPRSS5* | -1.14 | 0.82 | 0.98 | 0.71 | 0.24 | 0.83 | 0.72 | 0.76 | 39 | 16 | 5 | 1 |
| 3 | *EGR3* | 0.04 | 0.82 | 0.98 | 0.71 | 0.24 | 0.83 | 0.72 | 0.76 | 39 | 16 | 5 | 1 |
| 4 | *TP53* | 5.05 | 0.82 | 0.95 | 0.72 | 0.29 | 0.75 | 0.72 | 0.71 | 38 | 15 | 6 | 2 |
| 5 | *FOSB* | 1.15 | 0.82 | 1.00 | 0.69 | 0.14 | 1.00 | 0.70 | 0.86 | 40 | 18 | 3 | 0 |
| 6 | *NR4A1* | 3.22 | 0.81 | 1.00 | 0.68 | 0.10 | 1.00 | 0.69 | 0.90 | 40 | 19 | 2 | 0 |
| 7 | *SFTPB* | -0.10 | 0.80 | 0.95 | 0.69 | 0.19 | 0.67 | 0.69 | 0.81 | 38 | 17 | 4 | 2 |
| 8 | *IFI27* | 3.87 | 0.79 | 0.98 | 0.66 | 0.05 | 0.50 | 0.66 | 0.95 | 39 | 20 | 1 | 1 |
| 9 | *PLLP* | 5.66 | 0.78 | 0.95 | 0.66 | 0.05 | 0.33 | 0.64 | 0.95 | 38 | 20 | 1 | 2 |
| 10 | *CELA3A* | -3.84 | 0.78 | 0.95 | 0.66 | 0.05 | 0.33 | 0.64 | 0.95 | 38 | 20 | 1 | 2 |
| 11 | *ATF3* | 2.94 | 0.78 | 0.95 | 0.66 | 0.05 | 0.33 | 0.64 | 0.95 | 38 | 20 | 1 | 2 |
| 12 | *IGHV3-43* | -1.38 | 0.78 | 0.95 | 0.66 | 0.05 | 0.33 | 0.64 | 0.95 | 38 | 20 | 1 | 2 |
| 13 | *CDH1* | 8.61 | 0.78 | 0.95 | 0.66 | 0.05 | 0.33 | 0.64 | 0.95 | 38 | 20 | 1 | 2 |
| 14 | *PGC* | 2.48 | 0.74 | 0.88 | 0.64 | 0.05 | 0.17 | 0.59 | 0.95 | 35 | 20 | 1 | 5 |
| 15 | *GKN2* | -0.11 | 0.74 | 0.88 | 0.64 | 0.05 | 0.17 | 0.59 | 0.95 | 35 | 20 | 1 | 5 |
| 16 | *IGHV4-31* | -0.91 | 0.74 | 0.88 | 0.64 | 0.05 | 0.17 | 0.59 | 0.95 | 35 | 20 | 1 | 5 |
| 17 | *IGHV3-53* | -0.92 | 0.74 | 0.88 | 0.64 | 0.05 | 0.17 | 0.59 | 0.95 | 35 | 20 | 1 | 5 |
| 18 | *PNLIPRP1* | -2.73 | 0.72 | 0.83 | 0.63 | 0.10 | 0.22 | 0.57 | 0.90 | 33 | 19 | 2 | 7 |
