# Supplementary material for: EsoDetect: computational validation and algorithm development of a novel diagnostic and prognostic tool for dysplasia in Barrett’s esophagus
Source: PeerJ. 2025 Jul 3;13:e19613. doi: 10.7717/peerj.19613 (PMC12229151; doi:10.7717/peerj.19613)
Supplement: Supplemental Information 14 [file peerj-13-19613-s014.docx]

| **Gene** | **Count** | **Frequency (%)** |
| --- | --- | --- |
| *IGHV3-43* | 1115 | 100.00 |
| *SLC38A4* | 1097 | 98.39 |
| *PLLP* | 1072 | 96.14 |
| *CELA3A* | 923 | 82.78 |
| *IGHV4-31* | 850 | 76.23 |
| *TMPRSS5* | 646 | 57.94 |
| *TP53* | 645 | 57.85 |
| *NR4A1* | 633 | 56.77 |
| *ATF3* | 625 | 56.05 |
| *IFI27* | 581 | 52.11 |
| *PGC* | 530 | 47.53 |
| *GKN2* | 490 | 43.95 |
| *PNLIPRP1* | 470 | 42.15 |
| *SFTPB* | 460 | 41.26 |
| *CDH1* | 379 | 34.00 |
| *FOSB* | 369 | 33.10 |
| *EGR3* | 355 | 31.84 |
| *IGV3-53* | 330 | 29.60 |
