# Supplementary material for: EsoDetect: computational validation and algorithm development of a novel diagnostic and prognostic tool for dysplasia in Barrett’s esophagus
Source: PeerJ. 2025 Jul 3;13:e19613. doi: 10.7717/peerj.19613 (PMC12229151; doi:10.7717/peerj.19613)
Supplement: Supplemental Information 15 [file peerj-13-19613-s015.docx]

| **Gene** | **Count** | **Frequency (%)** |
| --- | --- | --- |
| *SNAI1* | 5545 | 95.70245 |
| *DUSP1* | 3837 | 66.22368 |
| *CEBPB* | 3791 | 65.42975 |
| *C1S* | 3672 | 63.37591 |
| *COL4A1* | 3409 | 58.83673 |
| *LAMC1* | 3157 | 54.4874 |
| *CEBPD* | 3121 | 53.86607 |
| *ZEB1* | 3105 | 53.58992 |
| *TWIST1* | 2940 | 50.74215 |
| *CCN1* | 2913 | 50.27615 |
| *TP53* | 2872 | 49.56852 |
| *ACTN1* | 2609 | 45.02934 |
| *VWF* | 2592 | 44.73593 |
| *PLPP3* | 2336 | 40.31757 |
| *CDH1* | 1140 | 19.67553 |
